# Model‐based reconstruction framework for correction of signal pile‐up and geometric distortions in prostate diffusion MRI

**DOI:** 10.1002/mrm.27547

**Published:** 2018-11-04

**Authors:** Muhammad Usman, Lebina Kakkar, Alex Kirkham, Simon Arridge, David Atkinson

**Affiliations:** ^1^ Centre for Medical Image Computing, Department of Computer Science University College London London United Kingdom; ^2^ Centre for Medical Imaging, Division of Medicine University College Hospital London United Kingdom; ^3^ Department of Radiology University College Hospital London United Kingdom

**Keywords:** geometric distortion, diffusion, prostate MRI, signal pile‐up

## Abstract

**Purpose:**

Prostate diffusion‐weighted MRI scans can suffer from geometric distortions, signal pileup, and signal dropout attributed to differences in tissue susceptibility values at the interface between the prostate and rectal air. The aim of this work is to present and validate a novel model based reconstruction method that can correct for these distortions.

**Methods:**

In regions of severe signal pileup, standard techniques for distortion correction have difficulty recovering the underlying true signal. Furthermore, because of drifts and inaccuracies in the determination of center frequency, echo planar imaging (EPI) scans can be shifted in the phase‐encoding direction. In this work, using a B_0_ field map and a set of EPI data acquired with blip‐up and blip‐down phase encoding gradients, we model the distortion correction problem linking the distortion‐free image to the acquired raw corrupted k‐space data and solve it in a manner analogous to the sensitivity encoding method. Both a quantitative and qualitative assessment of the proposed method is performed in vivo in 10 patients.

**Results:**

Without distortion correction, mean Dice similarity scores between a reference T2W and the uncorrected EPI images were 0.64 and 0.60 for b‐values of 0 and 500 s/mm^2^, respectively. Compared to the Topup (distortion correction method commonly used for neuro imaging), the proposed method achieved Dice scores (0.87 and 0.85 versus 0.82 and 0.80) and better qualitative results in patients where signal pileup was present because of high rectal gas residue.

**Conclusion:**

Model‐based reconstruction can be used for distortion correction in prostate diffusion MRI.

## 1 INTRODUCTION

Prostate cancer is the most commonly diagnosed cancer in males and the second cause of cancer‐related deaths in men.^1^ Early detection of prostate cancer can help in better management and treatment of disease when the cancer is still localized. Multiparametric prostate magnetic resonance imaging (mpMRI) is now becoming a common tool for early detection and staging of prostate cancer. Typically, mpMRI is done as a combination of T2‐weighted (T2W), diffusion‐weighted MRI (DWI), and dynamic contrast‐enhanced MRI (DCE) scans, which determine the likelihood of clinically significant cancer at a particular location within prostate.^2^, ^3^ DWI is the main sequence for cancer detection in the peripheral zone of the prostate,^4^ where 75% of tumors usually occur.^5^ Prostate cancers normally show abnormal diffusion restrictions and high signal in DWI images.

In DWI, single‐shot echo planar imaging (SS‐EPI) has been the preferred k‐space read out technique because of its fast speed and robustness against motion artefacts. However, SS‐EPI has a disadvantage of low bandwidth along the phase‐encoding direction, making it sensitive to inhomogeneities in the magnetic field. In MRI, spatial encoding is achieved by using magnetic field gradients, and the measured signal represents the Fourier transform of the object. In the presence of magnetic field inhomogeneities, an additional off‐resonance field (called B_0_ field) will add an additional component to the linear magnetic field gradient so that the position‐frequency relationship is changed. This results in signal stretching in areas within the image where the gradient of B_0_ field has the same polarity as the phase‐encoding gradient. Conversely, signal compression or *pileup* occurs in regions where the B_0_ field gradient direction is opposite to that of the phase‐encoding gradient. This results in multiple pixels along the phase‐encoding direction being merged into a single pixel. The main source of B_0_ field inhomogeneities is the susceptibility differences that arise at the interface between tissues of different susceptibilities. Larger local susceptibility differences will create a stronger off‐resonance field that will result in more‐severe signal pileup or stretching within the image. For DWI prostate images, susceptibility differences result in severe distortions and poor depiction of zonal anatomy.^6^ This can result in nondiagnostic images, errors in image interpretation, or a requirement for biopsy. Additionally, DWI images from prostate cancer patients with metallic hip prostheses^7^ may also suffer from susceptibility‐induced distortions across the whole prostate region. In addition to susceptibility‐induced distortions, a translational shift may occur along the phase‐encoding direction^8^ in the reconstructed EPI images attributed to drifts in center frequency of the magnet.

Several methods have been proposed to address the B_0_ distortion problem in EPI images. Some of these techniques correct distortions directly in the image space using a B_0_ field calculated from a separate dual‐echo gradient echo scan.^9^, ^10^, ^11^, ^12^ Having an accurate B_0_ field, these methods can generally correct for signal‐stretching artefacts in the images. However, using EPI data acquired with only 1 phase‐encoding direction may not provide sufficient information to correct for signal pile‐up artefacts. To address this issue, image‐registration–based reverse‐phase–encoded gradient methods have been proposed for brain functional MRI that acquire the same slice twice with opposite phase‐encoding gradient directions, resulting in 2 data sets, *blip‐up* and *blip‐down*.^8^, ^13^ Using the assumption that the signal pile‐up effects with blip‐up data set will correspond to the signal‐stretching effects in the same region with blip‐down data set and vice versa, these methods attempt to find the B_0_ field as a symmetrical displacement field by using image registration that will result in identical corrected images in the 2 phase‐encoding gradient directions. However, instead of solving the full inverse problem constrained to the acquired raw k‐space data, these methods perform image‐based optimization. This may result in an erroneous calculation of the B_0_ field attributed to the lack of a unique solution between the corresponding locations in the blip‐up and blip‐down images (especially in regions with severe signal pileup),^14^ leading to artifacts or blurring in the final images. Furthermore, in image‐based methods, the registration becomes more difficult when the image signal‐to‐noise ratio (SNR) is poor^15^, ^16^ and registration errors may lead to failure in distortion correction. By defining a forward model linking the distortion free image to the acquired corrupted raw k‐space data, an exact solution of the full inverse problem may be achieved by using the forward and conjugate transpose operators with a conjugate gradient scheme.^17^ Distortions attributed to B_0_ field inhomogeneities may be corrected directly during the reconstruction process using this full inverse problem solution, although a previous estimation of the B_0_ field is needed. The complex averaging used in the reconstruction avoids the bias from noise that can occur when magnitude images are combined.^18^, ^19^, ^20^, ^21^ This is likely to be especially beneficial for high b‐value DWI images with low SNR.^18^, ^20^, ^21^

In this work, we propose a model‐based reconstruction framework to solve the full inverse problem of prostate DWI distortion correction. By using the EPI raw k‐space data from acquisitions with blip‐up and blip‐down phase‐encoding gradients, we model the distortion correction problem linking the corrupted k‐space data to the corrected image and solve it in a manner analogous to sensitivity encoding (SENSE),^22^ using the conjugate gradient (CG) iterative method. To solve the issue of translational shifts attributed to the uncertainties in center frequency, we include the centre frequency offset correction as an initial unknown in our framework. In addition, a phase correction is also incorporated into our framework to avoid any phase cancellation issues that may arise because of small motion of the tissue within the diffusion‐encoding gradients. Results using the proposed method are compared in 10 patients against a neuroimaging distortion correction method based on reverse‐phase–encoding gradient, known as Topup.^8^

## 2 METHODS

### 2.1 Acquisition

The proposed framework acquires 2 EPI data sets (blip‐up and blip‐down) with opposite phase‐encoding gradient directions. A B_0_ scan is acquired as a separate dual‐echo gradient echo scan for estimation of the B_0 _field.

### 2.2 Reconstruction

With B_0_ field inhomogeneities, the corrupted k‐space $Y^{j}$ corresponding to *j* ^th^ coil (*j* = 1, 2,…,*J*; *J* being total number of coils) can be related to the undistorted image **x** by the following model (Equation (1)):

(1) $$Y^{j}\left(k,l\right)=\sum_{n=0}^{N-1}\sum_{m=0}^{M-1}C^{j}\left(m,n\right)x\left(m,n\right)e^{-i2\pi (\frac{\mathrm{mk}}{M}+\frac{\mathrm{nl}}{N})}e^{-i2\pi ({\Delta B}_{0}\left(m,n\right).t\left(k,l\right))}$$

where *m, n* are image coordinate indices, M, N are image dimensions, *k, l* are k‐space coordinate indices,* t(k, l)* is the sample time for location *(k, l)* in k‐space, Δ**B**_0_ is the B_0_ field in Hz that is estimated from a separate dual echo gradient echo scan, $C^{j}$ is the *j* ^th^ coil sensitivity, and *j* = 1, 2,..,*J*

In the presence of a center frequency offset (∆*f*_0_), the model in Equation 1 is modified as Equation (2):

(2) $$Y^{j}\left(k,l\right)=\sum_{n=0}^{N-1}\sum_{m=0}^{M-1}C^{j}\left(m,n\right)x\left(m,n\right)e^{-i2\pi (\frac{\mathrm{mk}}{M}+\frac{\mathrm{nl}}{N})}e^{-i2\pi (\left({\Delta B}_{0}\left(m,n\right)+\Delta f_{0}\right).t\left(k,l\right))}$$

In the above expression, the center frequency offset ∆*f*_0_ is assumed to be a constant in space. For a given ∆*f*_0_, translational shifts in image space are equal and opposite for opposite phase‐encoding gradient directions (e.g., positive shift for blip‐up and negative for blip‐down scans or vice versa).

### 2.3 Proposed framework

The proposed reconstruction framework has the following 3 steps.

#### 2.3.1 Step 1. Estimation of center frequency offset (∆f_0_)

Let $Y_{1}^{j}$ and $Y_{2}^{j}$ be the k‐space for blip‐up and blip‐down EPI scans for *j* ^th^ coil, respectively. Mathematically, we can write (Equation (3)):

$$Y_{1}^{j}\left(k,l\right)=\sum_{n=0}^{N-1}\sum_{m=0}^{M-1}C^{j}\left(m,n\right)x\left(m,n\right)e^{-i2\pi (\frac{\mathrm{mk}}{M}+\frac{\mathrm{nl}}{N})}e^{-i2\pi (\left({\Delta B}_{0}\left(m,n\right)+\Delta f_{0}\right).t_{1}\left(k,l\right))}$$

(3) $$Y_{2}^{j}\left(k,l\right)=\sum_{n=0}^{N-1}\sum_{m=0}^{M-1}C^{j}\left(m,n\right)x\left(m,n\right)e^{-i2\pi \left(\frac{\mathrm{mk}}{M}+\frac{\mathrm{nl}}{N}\right)}e^{-i2\pi (\left({\Delta B}_{0}\left(m,n\right)+\Delta f_{0}\right).t_{2}\left(k,l\right))}$$

where *t*_1_ and *t*_2_ are the acquisition times of the k‐space samples for blip‐up and blip‐down scans, respectively. Equation 3 can be summarized as Equation [Link]:

$$Y_{1}^{j}=E_{1}^{j}x$$

(4) $$Y_{2}^{j}=E_{2}^{j}x$$

where $E_{1}^{j}$ and $E_{2}^{j}$ are the encoding operators for blip‐up and blip‐down scans, respectively. The preliminary model‐based conjugate phase reconstructions ($x_{\mathrm{cp}1}$ and $x_{\mathrm{cp}2}$) from multicoil data are calculated as a multicoil combination, where the *j*th coil contribution is obtained by performing adjoint of encoding operators $E_{1}^{j}$ and $E_{2}^{j}$ onto the corresponding k‐space $Y_{1}^{j}$ and $Y_{2}^{j}$, respectively.

Mathematically, $x_{\mathrm{cp}1}$ and $x_{\mathrm{cp}2}$ are expressed as Equation [Link]:

$$x_{\mathrm{cp}1}=\sum_{j=1}^{J}{\left(E_{1}^{j}\right)}^{H}Y_{1}^{j}$$

(5) $$x_{\mathrm{cp}2}=\sum_{j=1}^{J}{\left(E_{2}^{j}\right)}^{H}Y_{2}^{j}$$

where${\left(\cdot \right)}^{\text{H}}$ is the Hermitian operator.

The optimal center frequency offset ∆*f*_0,*opt*_ is estimated as a parameter that maximizes the mutual information (MI) similarity measure^23^ between preliminary model‐based conjugate phase reconstructions of the blip‐up and blip‐down EPI data ($x_{\mathrm{cp}1}$ and $x_{\mathrm{cp}2}$) acquired with a b‐value of 0 s/mm^2^ by solving the following unconstrained problem (Equation (6)):

(6) $$\Delta f_{0,\mathrm{opt}}=arg_{\Delta f_{0}}\max\left(MI\left(x_{\mathrm{cp}1},x_{\mathrm{cp}2}\right)\right)$$

The B_0_ field is corrected by updating it with estimated optimal center frequency offset ∆f_0,opt_. The definition of Mutual Information function used in our method is described in Appendix I.

#### 2.3.2 Step 2. Phase correction

For diffusion‐weighted data (b‐value > 0 s/mm^2^), the phase of the blip‐up and blip‐down data in the image space might be different because of small physiological motion that may occur during the diffusion sensitization gradients. If data are combined from the 2 phase‐encoding directions without phase correction, this may result in phase cancellation leading to signal dropout in the final reconstructed image. To cater for this issue, we propose to calculate a phase correction ∆Ф obtained by taking the Hermitian inner product between the preliminary model‐based conjugate phase reconstructions ($x_{\mathrm{cp}1}$ and $x_{\mathrm{cp}2}$) of blip‐up and blip‐down EPI data using corrected B_0_ field (Equation (7)):

(7) $$\Delta \phi \left(m,n\right)=angle<x_{\mathrm{cp}\text{1}}\left(m,n\right),x_{\mathrm{cp}\text{2}}^{*}\left(m,n\right)>$$

where <.> indicates the inner product, (.)* denotes the complex conjugate, and* m, n* are image coordinate indices. In the above expression, the phase correction ΔФ varies in space because of the spatial variance of phases in $x_{\mathrm{cp}1}$ and $x_{\mathrm{cp}2}$. The raw k‐space data are corrected in phase by applying the phase correction ΔФ in the image space as follows.

With k‐space $Y_{2}^{j}$ selected as a reference, phase correction was applied to $Y_{1}^{j}$ by (1) first applying the inverse Fourier transform (*IFT*) to it, (2) multiplying by an exponential term containing the phase correction ΔФ to get the corrected image ${\tilde{y}}_{1}^{j}$, and (3) transforming back to k‐space by the Fourier transform (*FT*) to get a final phase‐corrected k‐space ${\tilde{Y}}_{1}^{j}$. Mathematically (Equation [Link]),

$$\text{Step a}:\;y_{1}^{j}\;\leftarrow IFT\leftarrow Y_{1}^{j}$$

$$\text{Step b}:\;{\tilde{y}}_{1}^{j}\;=\;y_{1}^{j}\exp\left(-i\Delta \phi \right)$$

(8) $$\text{Step c}:\;{\tilde{y}}_{1}^{j}\to FT\to {\tilde{Y}}_{1}^{j}$$

#### 2.3.3 Step 3. Model‐based reconstruction

The phase‐corrected multicoil k‐space data ${\tilde{Y}}_{1}$and ${\tilde{Y}}_{2}$ and the encoding operators **E**_1_ and **E**_2_ for blip‐up and blip‐down scans can be expressed mathematically by stacking data and encoding operators from all J coils (Equation (6)):

$${\tilde{Y}}_{1}={\left[{\tilde{Y}}_{1}^{1}\;{\tilde{Y}}_{1}^{2}\cdot \cdot \cdot \cdot {\tilde{Y}}_{1}^{J}\right]}^{\text{T}}\cdot \cdot \cdot \text{and}\cdot \cdot {\tilde{Y}}_{2}={\left[Y_{2}^{1}\;Y_{2}^{2}\cdot \cdot \cdot \cdot Y_{2}^{J}\right]}^{\text{T}}$$

(9) $$E_{1}={\left[E_{1}^{1}E_{1}^{2}\cdot \cdot \cdot \cdot E_{1}^{J}\right]}^{\text{T}}\cdot \cdot \cdot \cdot \text{and}\cdot \cdot \cdot \cdot E_{2}={\left[E_{2}^{1}E_{2}^{2}\cdot \cdot \cdot \cdot E_{2}^{J}\right]}^{\text{T}}$$

**Figure 1:**
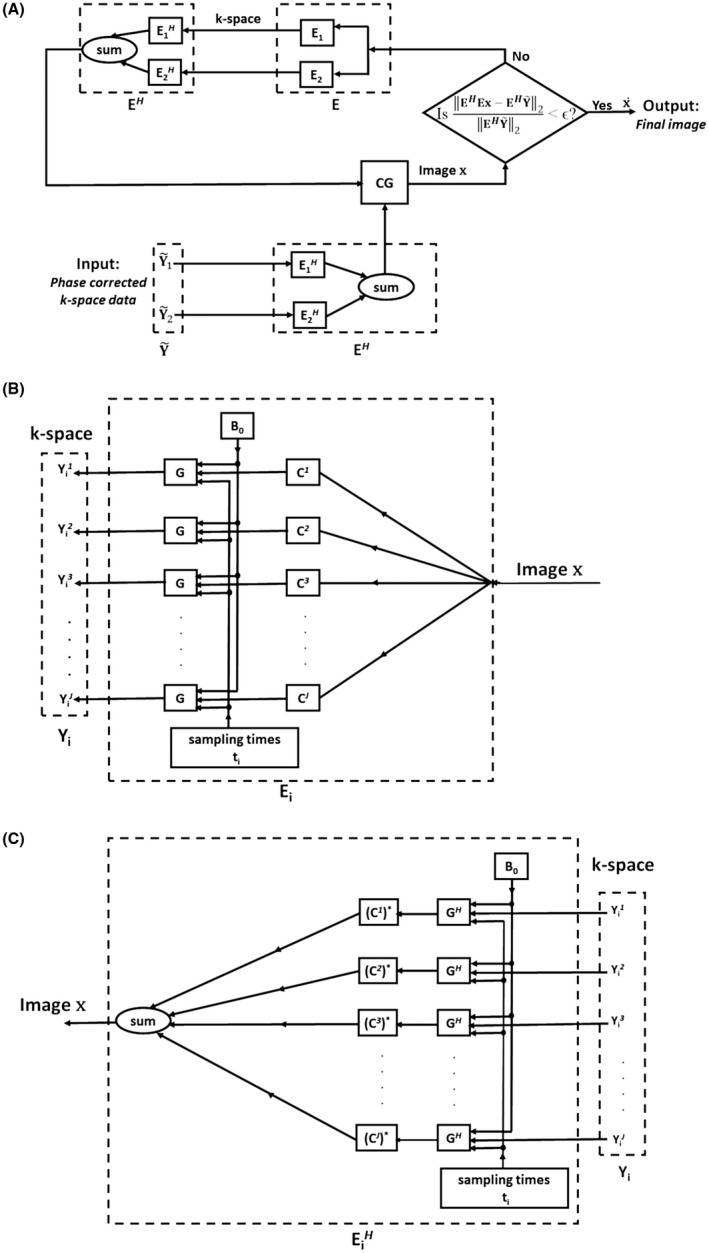
Implementation of iterative model‐based reconstruction (step 3 of proposed framework) using data from both blip‐up and blip‐down EPI scans. (a) Given the input k‐space data $\tilde{Y}={\left[{\tilde{Y}}_{1}{\tilde{Y}}_{2}\right]}^{\text{T}}$ that have been phase corrected in step 2, in each iteration of the conjugate gradient (CG) algorithm, we iterate back and forth between the k‐space and image **x** by encoding operator **E = [E**_1_ **E**_2_ **]** *^T^* and its adjoint **E*^H^*** that include the center frequency offset corrected B_0_ field. The convergence is achieved when the residual $r=\frac{E^{H}\mathrm{Ex}-E^{H}{\tilde{Y}}_{2}}{E^{H}{\tilde{Y}}_{2}}$ in the current iteration becomes smaller than ϵ (ϵ being a small number) giving the final distortion corrected output image $\dot{x}$. (b) Details of the forward encoding operator **E**_i _that takes the input single image **x** to the multicoil output k‐space **Y**_i_ for phase‐encoding direction i, i = 1 corresponds to the blip‐up and i = 2 corresponds to the blip‐down scans, respectively. The image **x** is first multiplied by the coil sensitivity **C** *^j^* (*j* = 1, 2,..,*J*). This is followed by a modified Fourier transform operator **G** that maps the product of image and coil sensitivity from image to Fourier space, taking into account the susceptibility effects determined by B_0_ field and k‐space sampling times t_i_, resulting in k‐space data $Y_{\text{i}}^{j}$ (*j* = 1, 2,..,*J*). (c) Details of the adjoint encoding operator $E_{\text{i}}^{H}$ that takes the multicoil k‐space data $Y_{\text{i}}^{j}$ (*j* = 1, 2,..,*J*) to single image **x** for phase‐encoding direction i, i = 1 corresponds to the blip‐up and i = 2 corresponds to the blip‐down scans, respectively. Each individual coil k‐space data $Y_{\text{i}}^{j}$ is transformed to image space by the adjoint of a modified Fourier transform operator (**G*^H^***). The resulting images are individually multiplied by complex conjugated coil sensitivities ${\left(C_{i}^{j}\right)}^{*}$ (*j* = 1, 2,..,*J*) and summed to give the final image **x**

Using the center frequency offset corrected Δ**B**_0 _obtained from step 1, for each b‐value and diffusion direction, the phase‐corrected EPI data ${\tilde{Y}}_{1}$ and ${\tilde{Y}}_{2}$ can be combined into a single formulation by setting ${\tilde{Y}=[\tilde{Y}}_{1}{\tilde{Y}}_{2}{]}^{\text{T}}$ and **E = [E**_1_ **E**_2_ **]^T^** in Equation (2). Model‐based reconstruction is done (Figure 1a) from combined k‐space data $\tilde{Y}$ in a manner analogous to an iterative SENSE reconstruction^22^ based on the conjugate gradient method by solving the following minimization problem (Equation (9)):

(10) $$min_{x}\left|\right|E^{\text{H}}\mathrm{Ex}-E^{\text{H}}\tilde{Y}{\left|\right|}_{2}^{2}$$

The above minimization problem is strictly convex because the encoding operator **E** is a linear function in **x.** Large negative local B_0_ field gradients in the phase‐encoding direction can make the encoding operator **E**_1_ or **E**_2_ singular or badly ill‐conditioned.^12^ The convergence of the CG iterations is achieved when the normalized residual $r=\frac{\left|\right|E^{H}\mathrm{Ex}-E^{H}\tilde{Y}{\left|\right|}_{2}}{\left|\right|E^{H}\tilde{Y}{\left|\right|}_{2}}$ ($\left|\right|\cdot {\left|\right|}_{2}$ denotes the l_2_ norm) in the current iteration becomes smaller than ϵ (ϵ being a small number) giving the final distortion corrected output image $\dot{x}$.

Figure 1b and Figure 1c shows visual illustrations for details of implementation of forward and adjoint encoding operators $E_{\text{i}}$ and $E_{\text{i}}^{H}$, respectively; i = 1 and i = 2 refer to blip‐up and blip‐down scans, respectively. The forward encoding operator $E_{\text{i}}$ involves multiplication of input image **x** by individual coil sensitivities **C** *^j^* that is followed by a modified Fourier transform operator **G** that maps the product of image and coil sensitivity from image to Fourier space, taking into account the susceptibility effects determined by B_0_ field and phase‐encoding direction dependent k‐space sampling times t_i_. The adjoint encoding operator $E_{\text{i}}^{H}$ involves transforming each individual coil k‐space data to image space by adjoint of modified Fourier transform operator (**G*^H^***) followed by multiplication by the corresponding complex conjugated coil sensitivity (**C** *^j^*)^*^ and the final summation over all the coils.

### 2.4 Experiments

Ten male patients (median weight, 84 [range, 68–98] kg and age 68 [57–79] years old) were recruited from the clinical prostate imaging pathway and were consented for additional image acquisitions. No antispasmodic agent was administered. Patients were placed feet first into the scanner and imaging was carried out during free breathing for all patients. The study was approved by the ethics committee, and written signed consent was obtained from all patients for the research scans.

Scanning was performed on a 3T scanner (Achieva; Philips Healthcare, Best, The Netherlands) equipped with 16 anterior + 16 posterior channel receive coil array. Single‐shot EPI data in both blip‐up and blip‐down phase‐encoding directions were acquired. The EPI scans had the following parameters: resolution = 2 × 2 × 4 mm^3^, field of view (FOV) = 180 to 220 × 180 to 220 × 55 to 90 mm^3^, partial Fourier acquisition with half scan factor of 0.75, TE/TR = 80 msec/2500 msec, phase‐encoding direction = anterior‐posterior (AP) axis with fat shift in the direction “P” for blip‐up and direction “A” for blip‐down scans, b‐values = 0 and 500 s/mm^2^, number of isotropic diffusion directions = 3, number of averages = 3, phase‐encode bandwidth per pixel = 10.4 to 11.2 Hz/pixel, and scan time = 30 sec. For calculation of the B_0_ field, a separate 3D dual‐echo gradient echo scan was acquired with the following parameters: resolution = 2 × 2 × 2 mm^3^, FOV = 200 to 250 × 200 to 250 × 70 to 120 mm^3^, flip angle = 6°, right to left phase‐encoding direction, SENSE acceleration factor = 1, TE difference (ΔTE) = 2.3 msec, TE1/TE2/TR = 4.6/6.9/8.7 msec, and scan time = 1 minute. For reference, axial T2W images were acquired using a turbo spin echo scan with the following parameters: resolution = 2 × 2 × 4 mm^3^, FOV = 180 to 220 × 180 to 220 × 55 to 90 mm^3^, SENSE acceleration factor = 2, TE/TR = 100/4700 msec, and scan time = 40 sec. Volume shimming was performed to cover the whole prostate and surrounding areas.

### 2.5 Data Postprocessing

To save the raw data together with the relevant information needed for the reconstruction framework, a software patch was implemented using ReconFrame software (Gyrotools Zurich, Zurich, Switzerland). EPI phase correction was performed using the ReconFrame tool to correct for ghosts originating from alternating offsets of phase encode lines in k‐space. Subsequent postprocessing was implemented in MATLAB (The MathWorks, Inc., Natick, MA).

The B_0_ field was calculated using the quantitative susceptibility mapping toolbox^24^ that estimates the field by a weighted least squares fit of temporally unwrapped phases in each voxel over echo time. A robust spline‐based smoothing^25^ was applied to the B_0_ field in image domain to smooth out noisy components. The 3D B_0_ field and T2W images were resampled to match the EPI scan resolution and the associated FOV. The iterative optimization in Equation 6 to estimate the optimal center frequency offset Δf_0,opt_ was carried out using the fminsearch function in MATLAB that uses the Nelder‐Mead simplex algorithm.^26^ The number of iterations in the algorithm was set to 10, and the optimal frequency offset Δf_0,opt_ was set to the value of Δ*f*_0 _found in the last iteration.

This was followed by phase correction (Equations 7 and 8) and model‐based reconstruction in Equation 10. The threshold *∊* for convergence of CG iterations in Equation 10 was set to 0.0025 in all our experiments. The model‐based corrected reconstruction was compared against uncorrected blip‐up and blip‐down reconstructions and the neuroimaging distortion correction Topup method.

### 2.6 Image Analysis

For quantitative evaluation, reconstructed images were converted to DICOM format and exported to Horos software,^27^ which is an open‐source medical image viewer. A region of interest (ROI) was manually drawn around the boundary of whole prostate in each T2W image and each reconstructed image by a radiologist with 12 years’ experience in reporting prostate MRI. Dice similarity coefficient scores^28^ were computed from the ROI overlap of each reconstruction and the reference T2W image. For qualitative assessment, the images reconstructed with different methods were put in a random order and blinded to method to avoid subjective assessment. The radiologist scored each image in terms of resolution, distortion, demarcation, and zonal anatomy.^29^ The “resolution” was defined as the ability to recognize detailed anatomical structures within the prostate, and it was assessed on a 5‐point scale (1 = poor; 2 = below average; 3 = average; 4 = above average; and 5 = excellent). The “distortion” was defined as the presence of artifacts, including signal pile‐up, signal dropout, warping, ghosting, and blurring. It was assessed on a 5‐point scale (1 = severe influence; 2 = significant influence; 3 = moderate influence; 4 = low influence; and 5 = no influence). The “demarcation” was defined as the ability to depict the prostatic capsule in a continuous fashion around the prostate. It was assessed on a 5‐point scale (1 = poor; 2 = below average; 3 = average; 4 = above average; and 5 = excellent). “Zonal anatomy” was defined as the ability to distinguish the transitional zone of the prostate from the peripheral zone and was assessed on a 5‐point scale (1 = poor; 2 = below average; 3 = average; 4 = above average; and 5 = excellent).

### 2.7 Phase correction procedures

The phase of DWI data (b‐value, >0 s/mm^2^) can change dynamically because of small physiological motion occurring during the diffusion sensitization gradients. To evaluate the performance of the phase correction procedure used in our proposed framework, a mid‐prostate single slice was acquired for b‐value of 500 s/mm^2 ^on 2 patients for 40 dynamics with blip‐up phase‐encoding gradient. Model‐based reconstruction was done with and without phase correction for each pair of dynamics (dynamic 1 and dynamic 2, dynamic 1 and dynamic 3,….) in each diffusion direction with dynamic 1 selected as a reference. The goal is to check whether the proposed phase correction can work in cases where the dynamics have significantly different phases from one another in the conjugate phase reconstructions.

## 3 RESULTS

### 3.1 Estimation of center frequency offset (∆f_0_)

**Figure 2:**
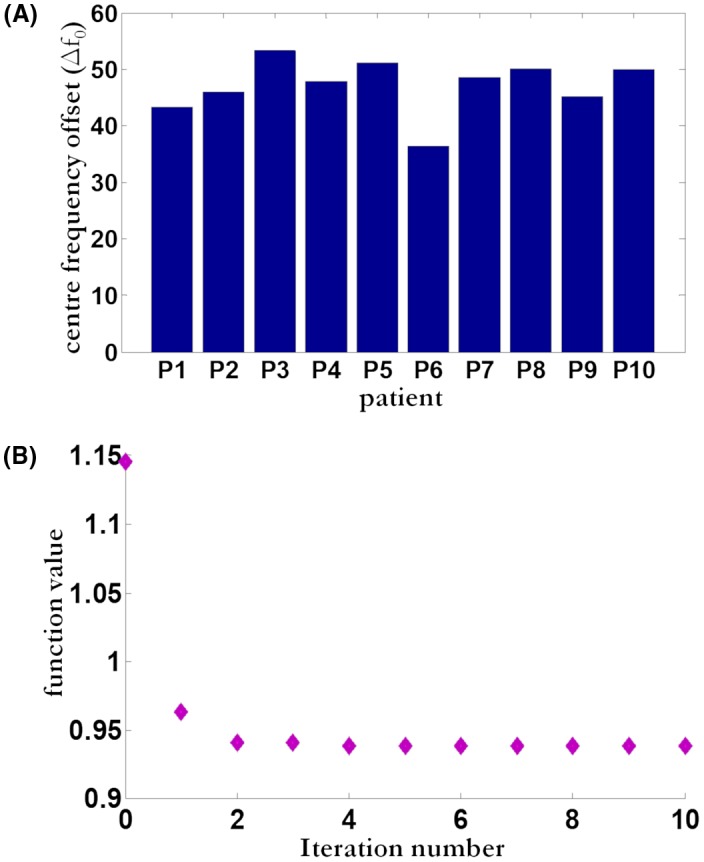
In vivo results. (a) Estimated center frequency offset ∆f_0_ (measured in Hz) in mid‐prostate slice as a function of patient number. (b) Convergence of mutual information‐based optimization as a function of iteration number

For all patients, the values of optimal center frequency offset (in Hz) for a mid‐prostate slice are shown in Figure 2a, together with a convergence plot of objective function (negative of mutual information function) as a function of iteration number in Figure 2b. The curve in Figure 2b shows that the objective function minimum (or equivalently mutual information function maximum) was achieved in 5 to 6 iterations. Mean frequency offset across all patients was 47.15 Hz with a standard deviation of 5.2 Hz.

### 3.2 Model‐based reconstruction

**Figure 3:**
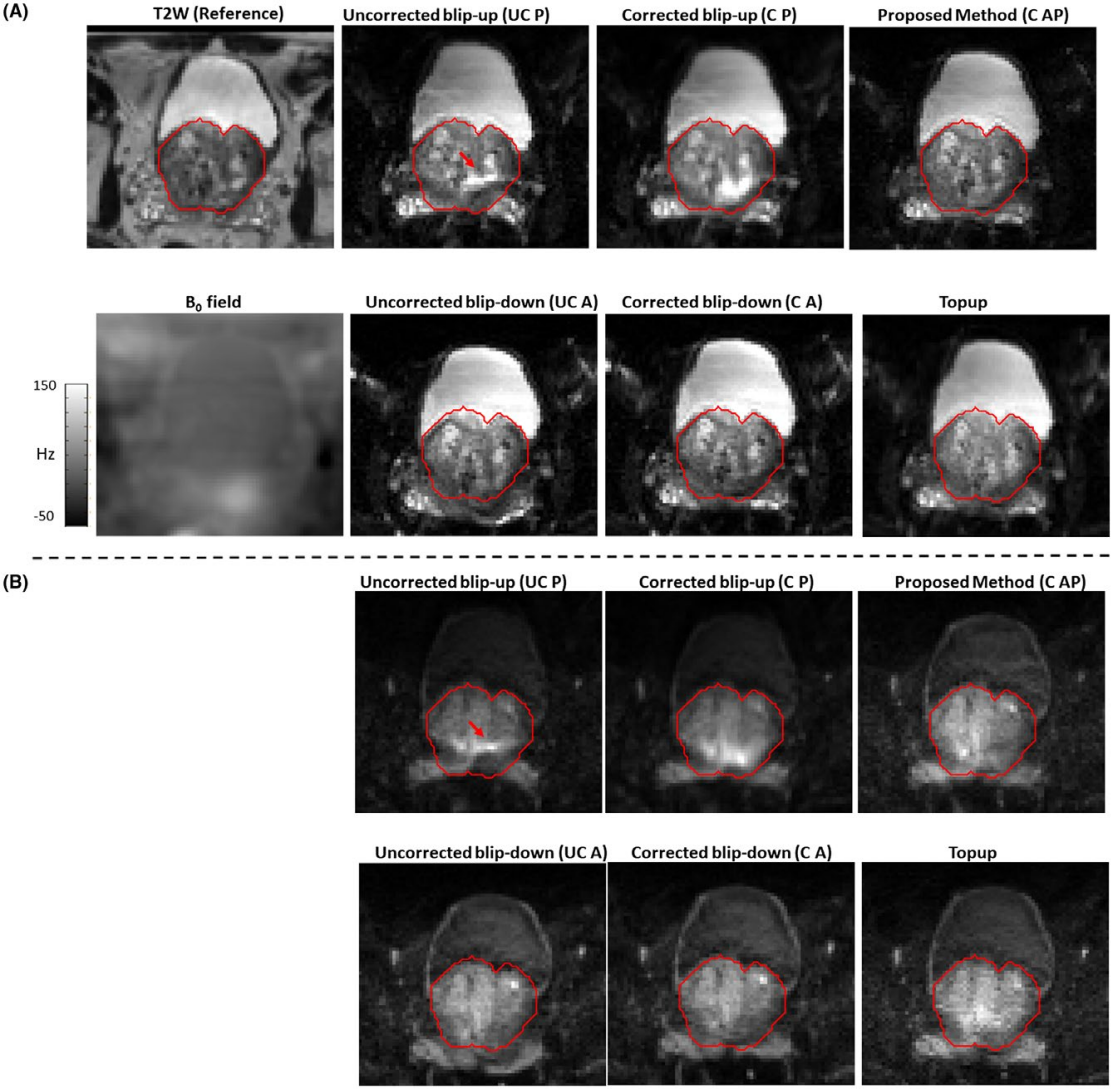
In vivo patient 1 reconstruction results for b‐value of 0 and b‐value of 500 s/mm^2^ are shown in (a) and (b), respectively. The reference T2W image and estimated B_0_ field (in Hz) are shown in (a). For EPI scans, the AP axis was selected as the phase‐encoding direction with fat shift in the direction “P” for blip‐up and “A” for blip‐down scans, respectively. Whole prostate (red) was delineated on reference T2W image and overlaid on reconstructions without distortion correction (uncorrected blip‐up [UC P] and uncorrected blip‐down [UC A]), reconstruction with distortion correction using data from 1 direction only (corrected blip‐up [C P] and corrected blip‐down [C A]), model‐based reconstruction using both blip‐up and blip‐down data (C AP), and Topup method (Topup). Red arrows indicate the regions of pile‐up. The proposed method corrected most of the signal pileup and has better resolution details than the Topup method

**Figure 4:**
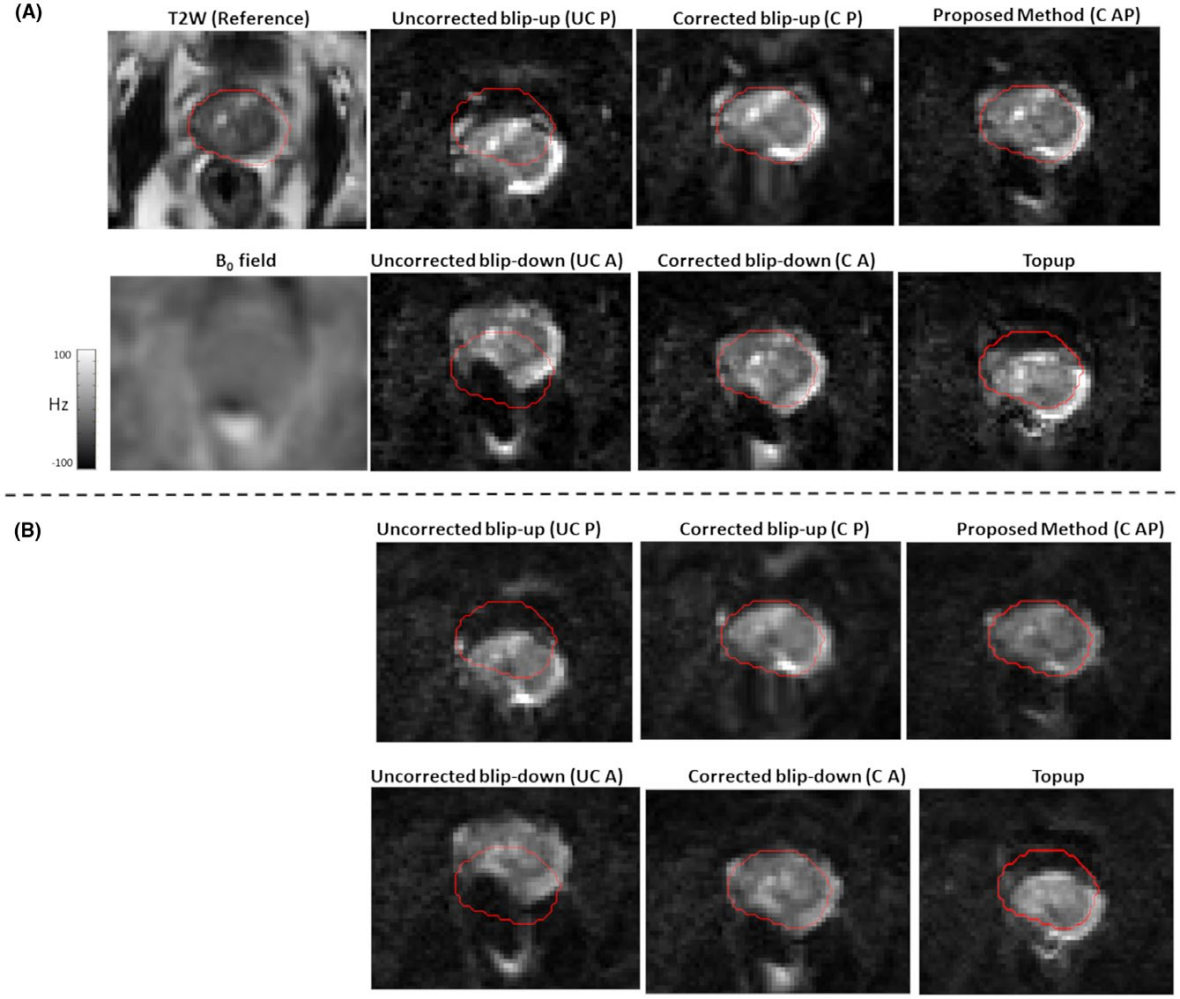
In vivo patient 2 reconstruction results for b‐value of 0 and b‐value of 500 s/mm^2^ are shown in (a) and (b), respectively. The reference T2W image and estimated B_0_ field (in Hz) are shown in (a). For EPI scans, the AP axis was selected as the phase‐encoding direction with fat shift in the direction “P” for blip‐up and “A” for blip‐down scans, respectively. Whole prostate (red) was delineated on reference T2W and overlaid on reconstructions without distortion correction (uncorrected blip‐up [UC P] and uncorrected blip‐down [UC A]), reconstruction with distortion correction using data from 1 direction only (corrected blip‐up [C P] and corrected blip‐down [C A]), model‐based reconstruction using both blip‐up and blip‐down data (C AP), and Topup method (Topup). The proposed method performed better than all other reconstructions in terms of distortion correction

**Figure 5:**
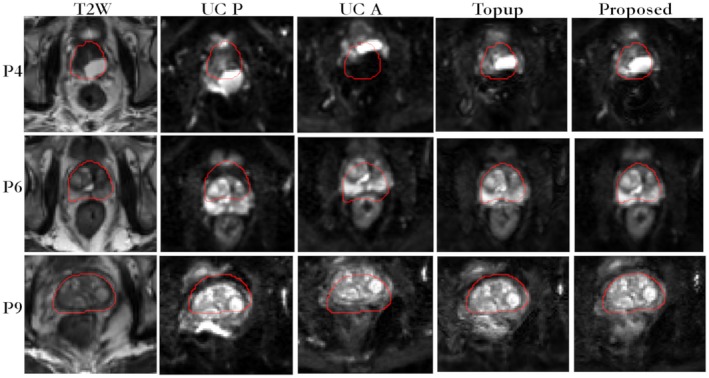
In vivo reconstruction results for selected patients (P4, P6, and P9) for data acquired at b‐value of 0 s/mm^2^. For EPI scans, the AP axis was selected as the phase‐encoding direction with fat shift in the direction “P” for blip‐up and “A” for blip‐down scans, respectively. Whole prostate (red) was delineated on a reference T2W image (left column) and overlaid on uncorrected blip‐up (UC P), uncorrected blip‐down (UC A), Topup, and proposed distortion‐corrected (C AP) reconstructions

**Figure 6:**
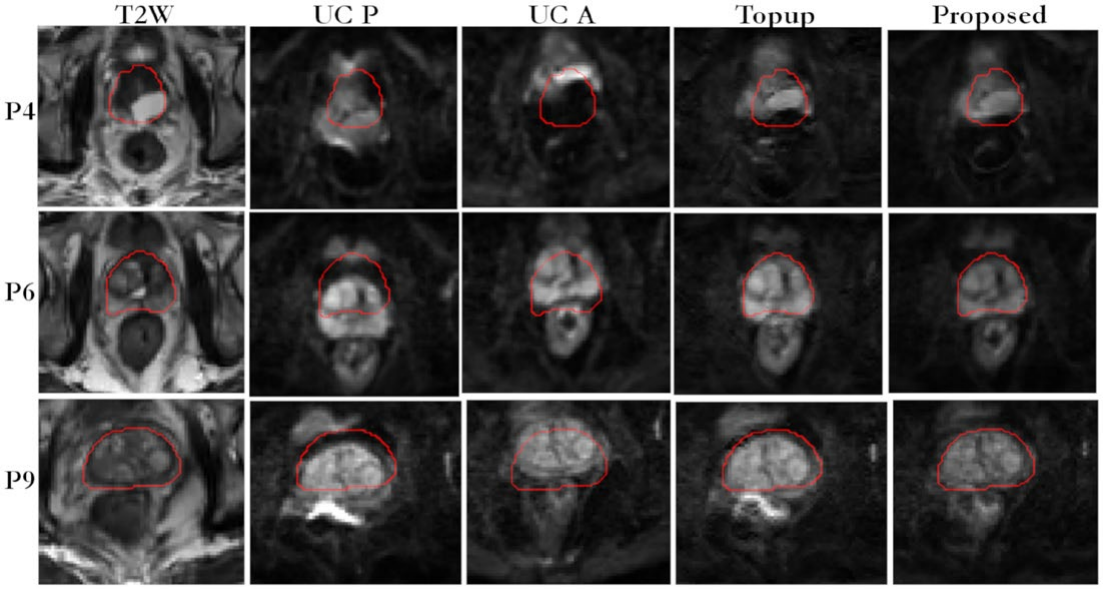
In vivo reconstruction results for selected patients (P4, P6, and P9) for data acquired at b‐value of 500 s/mm^2^. For EPI scans, the AP axis was selected as the phase‐encoding direction with fat shift in the direction “P” for blip‐up and “A” for blip‐down scans, respectively. Whole prostate (red) was delineated on a reference T2W image (left column) and overlaid on uncorrected blip‐up (UC P), uncorrected blip‐down (UC A), Topup, and proposed distortion‐corrected (C AP) reconstructions

In Figure 3, for patient 1, the reference T2W image, the B_0_ field, uncorrected blip‐up and blip‐down reconstructions, correction using blip‐up data only and blip‐down data only, and proposed model‐based reconstructions are shown for b‐values of 0 and 500 s/mm^2^, respectively. Because of susceptibility differences at the interface between different tissues in the prostate region, the variation in B_0_ was around 120 Hz that corresponds to a shift of 7 to 8 pixels at a bandwidth/pixel of ~15.80 Hz in phase‐encoding direction. This resulted in pileup in regions where the B_0_ field gradient magnitude was high and its direction was opposite to that of phase‐encoding gradient (see regions pointed out by red arrows in Figure 3). The reconstruction results for patient 2 are shown in Figure 4. The proposed method corrected for signal pile‐up artefacts that cannot be corrected using data from only 1 phase‐encoding gradient direction. For a mid‐prostate axial slice in a selective group of 3 patients (patient 4, patient 6, and patient 9), the proposed and Topup reconstructions for b‐values of 0 and 500 s/mm^2^ are shown in Figures 5 and 6, respectively. The complete set of results for patient 3 to patient 10 for b‐value of 0 and 500 s/mm^2 ^can be seen in Supporting Information Figures S1 and S2, respectively. Supporting Information Figure S3 shows an example plot of normalized residual error r as a function of CG iteration number. From our empirical observation, convergence of the CG method was achieved in 10 to 15 iterations.

**Figure 7:**
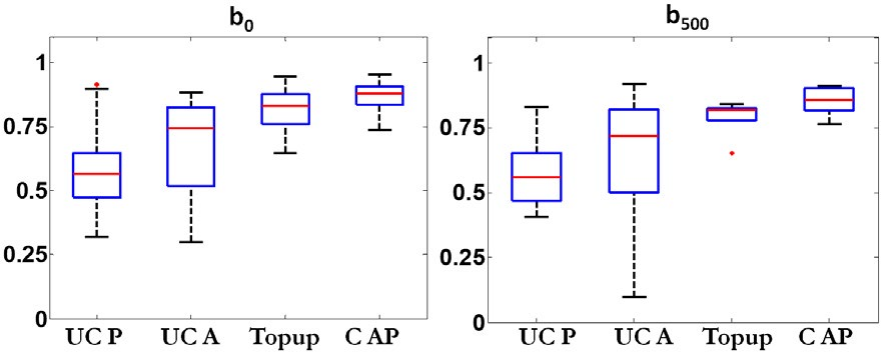
Quantitative assessment: box plots showing Dice scores (range, 0–1) for uncorrected blip‐up (UC P), uncorrected blip‐down (UC A), Topup, and proposed method (C AP) corresponding to b‐value of 0 s/mm^2^ (b_0_) and b‐value of 500 s/mm^2^ (b_500_) in the left and right columns, respectively. The T2W image was taken as a reference for calculation of Dice scores

Box plots showing the Dice scores for different reconstruction methods are shown in Figure 7. Mean Dice similarity scores were 0.60 (b‐value of 0) and 0.58 (b‐value of 500 s/mm^2^) for uncorrected blip‐up reconstructions and 0.68 (b‐value of 0) and 0.62 (b‐value of 500 s/mm^2^) for uncorrected blip‐down reconstructions. The proposed method achieved mean Dice similarity scores of 0.87 and 0.85 for b‐value of 0 and 500 s/mm^2^, respectively, and scored higher than the corresponding Dice similarity scores of the Topup method (0.82 and 0.80 for b‐value of 0 and 500 s/mm^2^, respectively). Qualitative scores (distortion, resolution, demarcation, and zonal anatomy) for all 4 methods corresponding to b‐value of 0 s/mm^2^ and b‐value of 500 s/mm^2 ^are given in Tables 1 and 2, respectively. Mean percentage of improvement in qualitative scores using the proposed method compared to other reconstructions is shown in Supporting Information Figure S4. The proposed method performed better, on average, than all other reconstructions for each qualitative measure.

**Table 1:** Qualitative scores across 10 patients for different reconstruction methods for data acquired at b‐value of 0 s/mm^2^

Quantity	Uncorrected blip‐up	Uncorrected blip‐down	Topup	Proposed method
Distortion	3.00 ± 0.67	3.30 ± 0.67	3.40 ± 0.51	3.50 ± 0.52
Resolution	3.20 ± 0.63	3.00 ± 0.67	3.10 ± 0.57	3.40 ± 0.69
Demarcation	3.10 ± 0.73	3.10 ± 0.73	3.00 ± 0.81	3.40 ± 0.51
Zonal anatomy	3.20 ± 0.63	3.30 ± 0.82	3.10 ± 0.31	3.60 ± 0.51

The associated standard deviations are also indicated.

**Table 2:** Qualitative scores across 10 patients for different reconstruction methods for data acquired at b‐value of 500 s/mm^2^

Quantity	Uncorrected blip‐up	Uncorrected blip‐down	Topup	Proposed method
Distortion	3.30 ± 0.67	3.40 ± 0.70	3.50 ± 0.52	3.90 ± 0.57
Resolution	3.00 ± 0.47	3.10 ± 0.74	3.00 ± 0.47	3.20 ± 0.78
Demarcation	3.30 ± 0.67	3.30 ± 0.48	3.50 ± 0.84	3.50 ± 0.52
Zonal anatomy	3.10 ± 0.56	2.80 ± 0.63	3.10 ± 0.56	3.10 ± 0.73

The associated standard deviations are also indicated.

### 3.3 Phase correction

**Figure 8:**
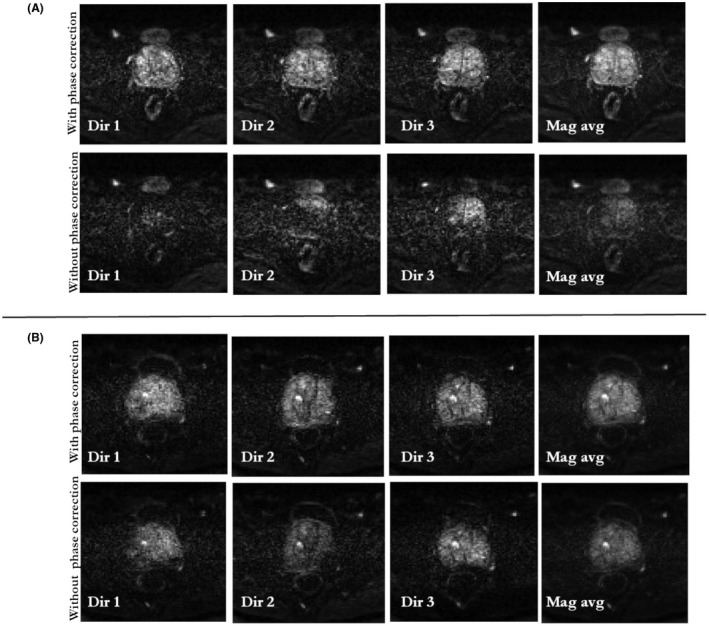
Evaluation of phase correction procedure: model‐based reconstruction with and without phase correction for dynamic frames of data acquired at b‐value of 500 s/mm^2^. Reconstructions for each individual diffusion direction (Dir 1, Dir 2, and Dir 3) are shown for patient 11 and patient 1 in (a) and (b), respectively. Data are combined from 2 dynamics (dynamic 1 and 13 for patient 11, dynamic 1 and 25 for patient 1) that had significantly different phases in conjugate phase reconstructions. Without phase correction, the phase cancellation in each model‐based reconstruction leads to signal dropout and signal cancellations (bottom row in a and b). The proposed phase correction procedure preserved signal in each diffusion direction (top row in a and b). The magnitude average (Mag avg) of model‐based reconstructions from the 3 directions (Dir 1, Dir 2, and Dir 3) is shown in the right column

Figure 8 shows the usefulness of phase correction used in the proposed method for patient 11 and patient 1 for data combined from a pair of dynamics acquired with diffusion weighting (b‐value of 500 s/mm^2^). Here, results are shown for a pair of dynamics (dynamic 1 and 13 for patient 11, dynamic 1 and 25 for patient 1) that had significant different phases in conjugate phase reconstructions. Without phase correction, signal cancellation occurred in the reconstructed image in each diffusion direction. By using the proposed phase correction (Equation 8), the signal was preserved in each diffusion direction. The magnitude average of the reconstructions in three diffusion directions (Mag Avg) is shown in the right column that had close correspondence to the reference T2W image.

## 4 DISCUSSION

A novel model‐based reconstruction framework is proposed that can correct for geometric distortions, signal pileup, and signal dropout in diffusion‐weighted prostate images. By using the power of complimentary encoding information within data from both blip‐up and blip‐down directions, the model‐based framework was able to correct most of the pileup in regions of severe distortions and performed better than the Topup method (commonly used for neuroimaging) or model‐based reconstructions using data from 1 phase‐encoding gradient direction only. The proposed mutual information maximization used for correction of center frequency offset in the B_0_ field may also be used as a method for overcoming uncertainties in coordinates that may happen in the process of reconstruction from raw data.

The proposed method assumes the B_0_ field to be static except for center frequency offset attributed to the frequency drift between B_0_ and EPI scans. In case of motion or changes in the rectal area adjacent to the prostate region between the EPI and B_0_ scans, the B_0_ field estimated from B_0_ scan may be mismatched, resulting in inaccurate distortion correction. Some of the remaining distortions in the proposed method reconstructions can be attributed to dynamic changes in B_0_ field because no antispasmodic agent drug was administered in our scans that would suppress bowel movements and/or rectal gas. Changes in B_0_ might be addressed by a joint estimation of both B_0_ and the corrected EPI images, starting with the initial B_0_ fields estimated from B_0 _scan.^30^

For higher b‐values (b‐value, > = 1000 s/mm^2^), the low SNR in the reconstructed images might affect the inverse problem and the technique may benefit from preconditioning or additional regularization.

Our proposed method uses a B_0_ field estimated from a dual‐echo gradient echo scan. In image regions where the individual echoes have low SNR or missing signal (especially in the rectal‐air region), the measurement of B_0_ field in that area might not be always possible, leading to noisy and unwrapped phases in the B_0_ field. To address this issue, B_0_ field might be modeled in this region using a susceptibility map distribution^31^ that could be obtained from a segmentation of air and tissue areas on a reference T2W image. Alternatively, a projection onto dipole fields (PDF) method^32^ might also be used for estimating the B_0_ field inside the rectal‐air region. The PDF method calculates the B_0_ field inside an ROI by projection of known dipole fields from outside the ROI.

Last, the proposed method does not include any physiological motion effects that may occur between the EPI blip‐up and blip‐down scans. In case of motion between blip‐up and blip‐down scans, both motion parameters and the image would need to be estimated simultaneously and the optimization in Equation 10 becomes nonconvex. The optimization may be simplified by estimating motion in a preceding step similar to the framework in a previous work^33^ by registering model‐based reconstructions from blip‐up and blip‐down scans with either blip‐up or blip‐down reconstruction being set as the reference. The estimated motion fields can then be incorporated into Equation 10 by motion matrix transformation^17^, ^33^ that will relate the acquired corrupted k‐space data to corrected images through a combined model that is a concatenation of B_0_ distortion and motion corruption effects.

Prostate diffusion MRI is recognized as a potential biomarker for tumor detection, but, currently, it is unusable in some patients because of significant distortions. We proposed a novel model‐based reconstruction framework that can correct these distortions by using data from opposite phase‐encoding gradient directions. The proposed method was applied successfully in 10 clinical patients, despite no antispasmodic drug being administered. The proposed technique may offer potential to radiologists and clinicians by increasing the diagnostic value of prostate images for tumor detection, thus making prostate MRI a more reliable and reproducible biomarker in future.

## Supporting information


**FIGURE S1** In vivo reconstruction results for patients 3 (P3) to patient 10 (P10) for data acquired at b‐value of 0. For EPI scans, the AP axis was selected as the phase‐encoding direction with fat shift in the direction “P” for blip‐up and “A” for blip‐down scans, respectively. Whole prostate (red) was delineated on reference T2W image (left column) and overlaid on uncorrected blip‐up (UC P), uncorrected blip‐down (UC A), Topup, and proposed distortion‐corrected (C AP) reconstructions
**FIGURE S2** In vivo reconstruction results for patients 3 (P3) to patient 10 (P10) for data acquired at b‐value of 500 s/mm^2^. For EPI scans, the AP axis was selected as the phase‐encoding direction with fat shift in the direction “P” for blip‐up and “A” for blip‐down scans, respectively. Whole prostate (red) was delineated on reference T2W image (left column) and overlaid on uncorrected blip‐up (UC P), uncorrected blip‐down (UC A), Topup, and proposed distortion‐corrected (C AP) reconstructions
**FIGURE S3** Plot of normalized residual error $r=\frac{E^{H}\mathrm{Ex}-E^{H}{\tilde{Y}}_{2}}{E^{H}{\tilde{Y}}_{2}}$ as a function of the CG iteration number
**FIGURE S4** Qualitative assessment: mean percentage of improvement in qualitative scores (distortion, resolution, demarcation, and zonal anatomy) using model‐based reconstruction compared to uncorrected blip‐up (UC P), uncorrected blip‐down (UC‐A), and Topup methods. The results are shown for b‐values of 0 and 500 s/mm^2^. The improvements in all the qualitative scores for the proposed method were positive compared to the other reconstructions

## APPENDIX 1

The mutual information function MI(A,B)^23^ between the 2 images A and B, is defined as:

$$\text{MI}\left(\text{A},\text{B}\right)=\text{H}\left(\text{A}\right)+\text{H}\left(\text{B}\right)-\text{H}\left(\text{A},\text{B}\right)$$

where H(A) and H(B) are marginal entropy functions for images A and B, respectively, given as:

$$\text{H}\left(\text{A}\right)=-\sum_{a}P_{a}logP_{a}\cdot \cdot \cdot \cdot \text{and}\cdot \cdot \cdot \cdot \text{H}\left(\text{B}\right)=-\sum_{b}P_{b}logP_{b}$$

*P_a_* being the probability of pixels in image A having signal intensity *a* and *P_b_* being the probability of pixels in image B having signal intensity *b*.

H(A, B) is the joint entropy function of images A and B calculated as:

$$\text{H}\left(\text{A},\text{B}\right)=-\sum_{a}\sum_{b}P_{\mathrm{ab}}logP_{\mathrm{ab}}$$

$P_{\mathrm{ab}}$ being the joint probability of pixels in image A having intensity a and pixels in image B having intensity b.
