# Supplementary material for: Model‐based reconstruction framework for correction of signal pile‐up and geometric distortions in prostate diffusion MRI
Source: Magn Reson Med. 2018 Nov 4;81(3):1979–92. doi: 10.1002/mrm.27547 (PMC6492108; doi:10.1002/mrm.27547)
Supplement: Supplementary file 1 — FIGURE S1 In vivo reconstruction results for patients 3 (P3) to patient 10 (P10) for data acquired at b‐value of 0. For EPI scans, the AP axis was selected as the phase‐encoding direction with fat shift in the direction “P” for blip‐up and “A” for blip‐down scans, respectively. Whole prostate (red) was delineated on reference T2W image (left column) and overlaid on uncorrected blip‐up (UC P), uncorrected blip‐down (UC A), Topup, and proposed distortion‐corrected (C AP) reconstructions FIGURE S2 In vivo reconstruction results for patients 3 (P3) to patient 10 (P10) for data acquired at b‐value of 500 s/mm2. For EPI scans, the AP axis was selected as the phase‐encoding direction with fat shift in the direction “P” for blip‐up and “A” for blip‐down scans, respectively. Whole prostate (red) was delineated on reference T2W image (left column) and overlaid on uncorrected blip‐up (UC P), uncorrected blip‐down (UC A), Topup, and proposed distortion‐corrected (C AP) reconstructions FIGURE S3 Plot of normalized residual error r=EHEx-EHY~2EHY~2 as a function of the CG iteration number FIGURE S4 Qualitative assessment: mean percentage of improvement in qualitative scores (distortion, resolution, demarcation, and zonal anatomy) using model‐based reconstruction compared to uncorrected blip‐up (UC P), uncorrected blip‐down (UC‐A), and Topup methods. The results are shown for b‐values of 0 and 500 s/mm2. The improvements in all the qualitative scores for the proposed method were positive compared to the other reconstructions [file MRM-81-1979-s001.docx]

**“Model based Reconstruction Framework for Correction of Signal Pile-up and Geometric Distortions in Prostate Diffusion MRI” Supporting Information**


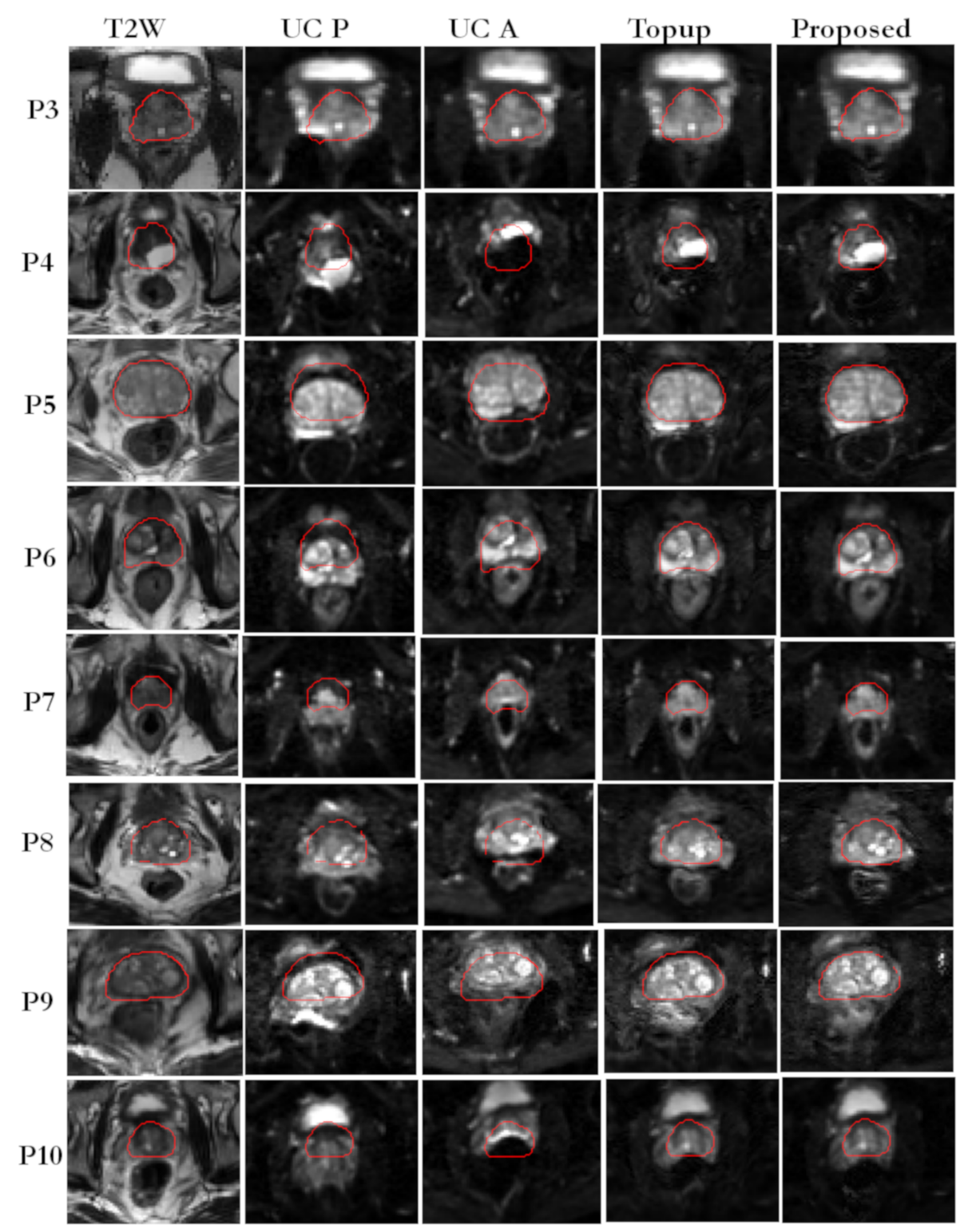


**Supporting Information Figure S1.** In-vivo reconstruction results for patients 3 (P3) to patient 10 (P10) for data acquired at b-value of 0. For EPI scans, the Anterior-Posterior (AP) axis was selected as the phase encoding direction with fat shift in the direction ‘P’ for blip-up and ‘A’ for blip-down scans, respectively. Whole prostate (red) was delineated on reference T2-weighted image (left column) and overlaid on uncorrected blip-up (UC P), uncorrected blip-down (UC A), Topup and proposed distortion corrected (C AP) reconstructions

**
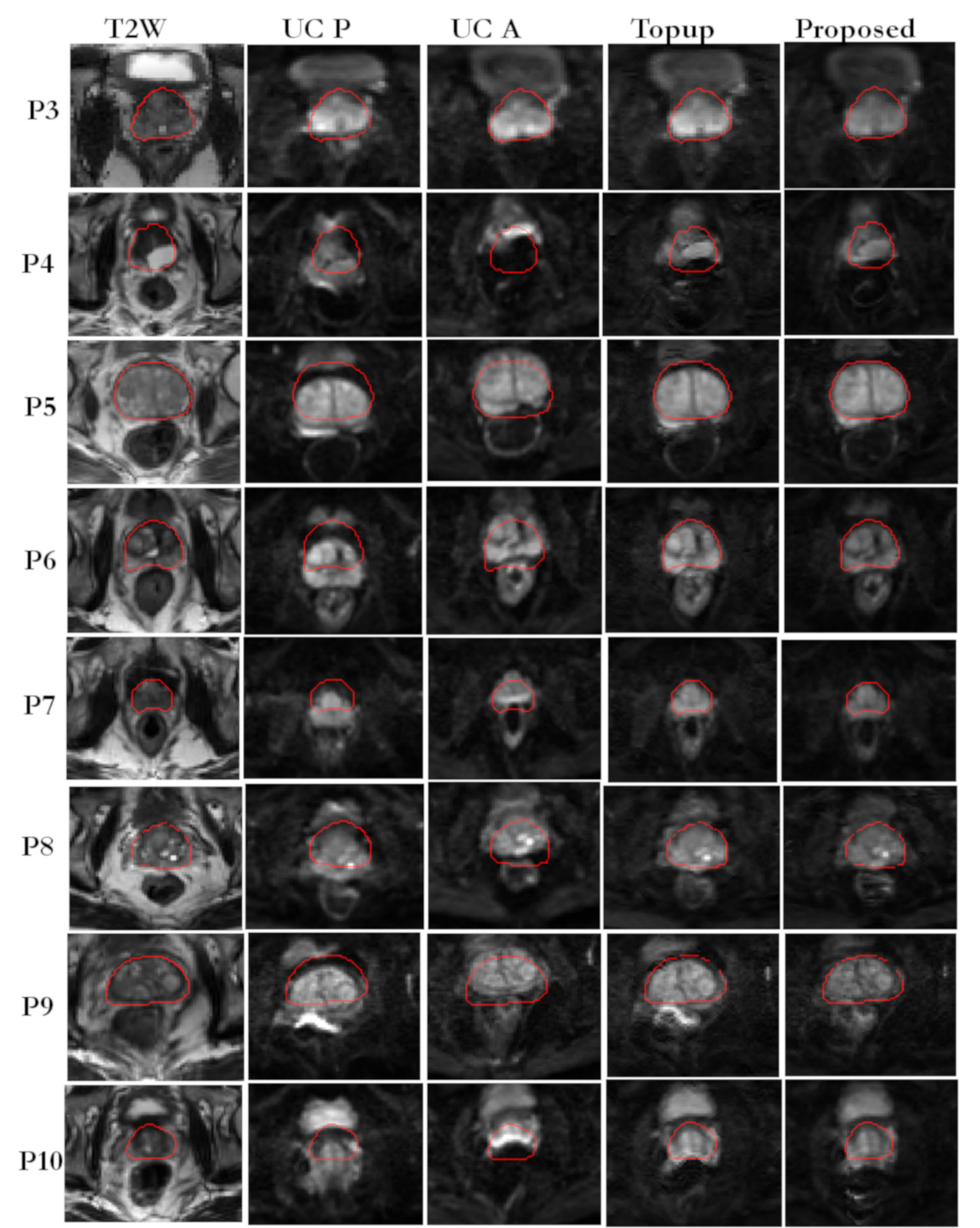
**

**Supporting Information Figure S2.** In-vivo reconstruction results for patients 3 (P3) to patient 10 (P10) for data acquired at b-value of 500 s/mm^2^. For EPI scans, the Anterior-Posterior (AP) axis was selected as the phase encoding direction with fat shift in the direction ‘P’ for blip-up and ‘A’ for blip-down scans, respectively. Whole prostate (red) was delineated on reference T2-weighted image (left column) and overlaid on uncorrected blip-up (UC P), uncorrected blip-down (UC A), Topup and proposed distortion corrected (C AP) reconstructions


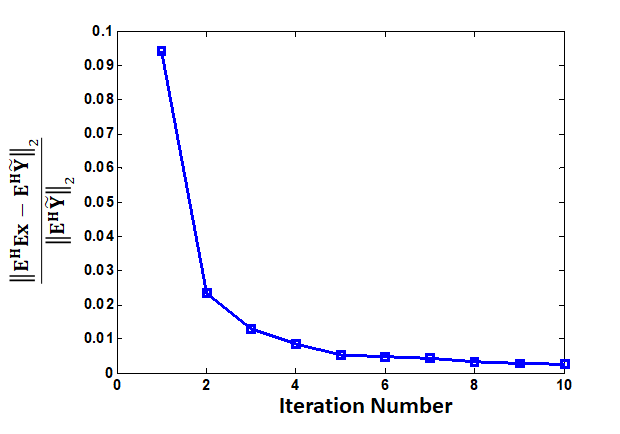


**Supporting Information Figure S3.** Plot of normalized residual error *r*=$\frac{\left\| \mathbf{E}^{\boldsymbol{H}}\mathbf{Ex-}\mathbf{E}^{\boldsymbol{H}}\tilde{\mathbf{Y}} \right\|_{2}}{\left\| \mathbf{E}^{\boldsymbol{H}}\tilde{\mathbf{Y}} \right\|_{2}}$ as function of CG iteration number


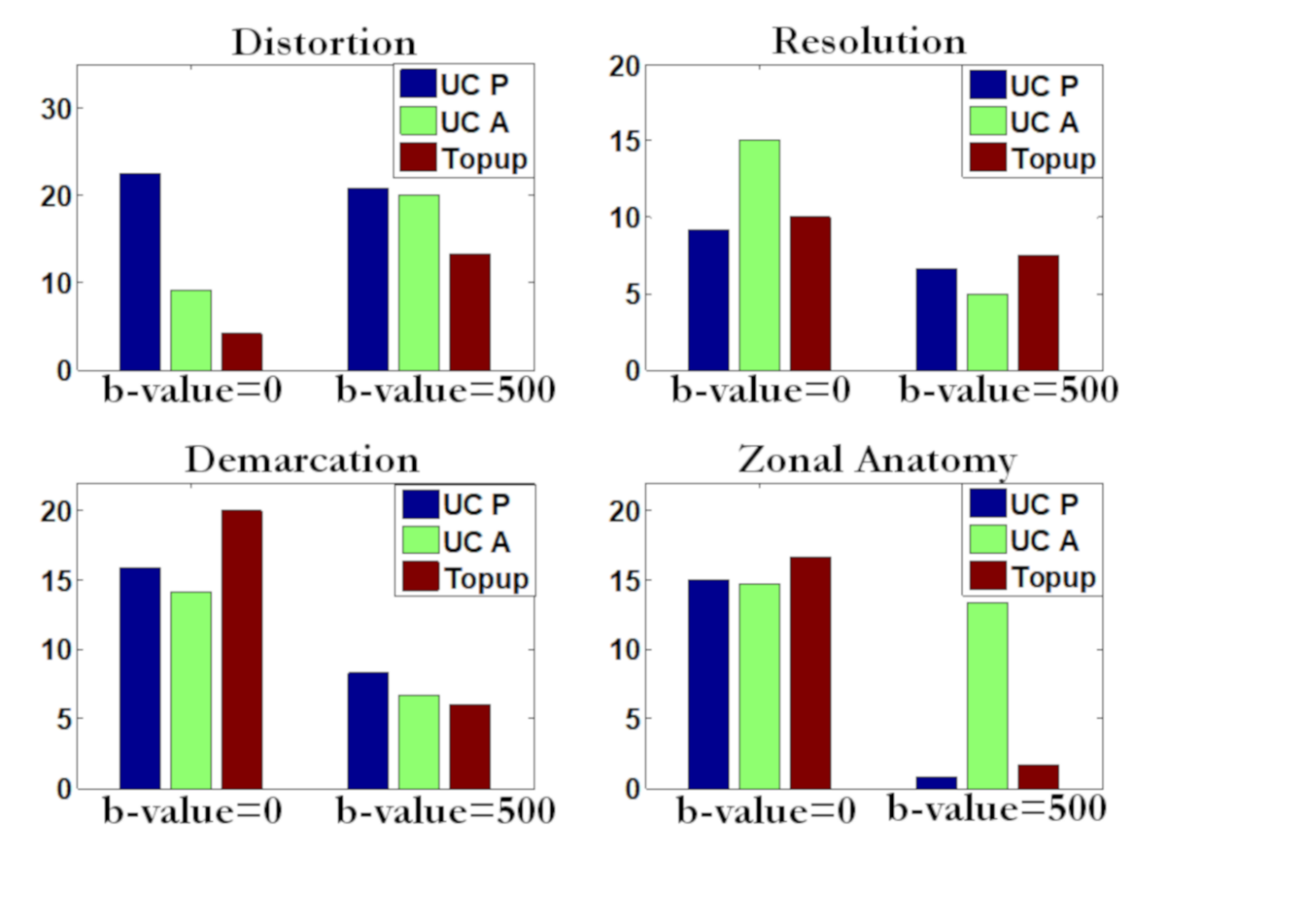
 **Supporting Information Figure S4. Qualitative assessment:** Mean percentage of improvement in qualitative scores (distortion, resolution, demarcation and zonal anatomy) using Model based reconstruction compared to Uncorrected blip-up (UC P), Uncorrected blip-down (UC-A) and Topup methods. The results are shown for b-values of 0 and 500 s/mm^2^. The improvements in all the qualitative scores for the proposed method were positive compared to the other reconstructions
